# Supplementary material for: Evidence for an energetic trade-off model linking inflammaging and immunosenescence in the US Health and Retirement Study and UK Biobank
Source: bioRxiv. 2025 Oct 17:2025.10.17.682903. Preprint. [Version 1] doi: 10.1101/2025.10.17.682903 (PMC12632893; doi:10.1101/2025.10.17.682903)
Supplement: Supplement 1 [file media-1.pdf]

Supplementary Materials

Table S1. Correlations among cytokines in the US HRS (n = 8,261)

	IL-6	TNFR1
TNFR1	0.42	
IL-10	0.35	0.44

All $p < 0.001$

Table S2. Correlations among cytokines in the UKB (n = 40,638)

	IL-6	TNFR1	IL-10	IL-1β	TNF-α
TNFR1	0.40				
IL-10	0.12	0.15			
IL-1β	0.08	0.14	0.04		
TNF-α	0.29	0.45	0.24	0.16	
GDF15	0.35	0.53	0.08	0.06	0.34

All $p < 0.001$

Table S3. HRS pro-inflammatory cytokine associations (n = 8,261)

	IL-6	TNFR1
Age (years)	0.015*** (0.001)	0.033*** (0.001)
Male (0,1)	0.038 (0.020)	-0.020 (0.018)
Hypertension or CVD (0,1)	0.255*** (0.022)	0.293*** (0.019)
Diabetes (0,1)	0.233*** (0.024)	0.322*** (0.021)
Cancer (0,1)	0.094*** (0.027)	0.130*** (0.024)

*** $p < 0.001$

Table S4. UKB pro-inflammatory cytokine associations (n = 40,638)

	IL-6	TNFR1	IL-1β	TNF-α
Age (years)	0.018*** (0.001)	0.026*** (0.001)	0.001* (0.001)	0.017*** (0.001)
Male (0,1)	-0.018* (0.009)	0.129*** (0.009)	-0.063*** (0.009)	-0.053*** (0.008)
Hypertension or CVD (0,1)	0.264*** (0.010)	0.262*** (0.010)	0.072*** (0.011)	0.151*** (0.009)
Diabetes (0,1)	0.253*** (0.021)	0.387*** (0.021)	0.010 (0.022)	0.197*** (0.019)
Cancer (0,1)	0.076*** (0.017)	0.099*** (0.017)	-0.0002 (0.018)	0.037* (0.016)

* $p < 0.05$; ** $p < 0.01$; *** $p < 0.001$

Table S5. Regression models predicting IL-10 and Naïve T cells in the US HRS, adjusting for CMV antibodies. Cytokines, T cell counts, and CMV antibodies were standardized, with resulting regression coefficients reflecting changes in standard deviations (n = 8,261). Estimates are reported as β (SE).

	IL-10	IL-10	CD4T	CD4T	CD8T	CD8T
Age (years)	-0.003* (0.001)	-0.003* (0.001)	-0.015*** (0.001)	-0.013*** (0.001)	-0.034*** (0.001)	-0.034*** (0.001)
Male (0,1)	0.098*** (0.018)	0.097*** (0.018)	-0.358*** (0.021)	-0.390*** (0.021)	-0.310*** (0.017)	-0.302*** (0.017)
Hypertension or CVD (0,1)	0.026 (0.020)	0.027 (0.020)	-0.027 (0.024)	-0.019 (0.023)	0.003 (0.020)	0.001 (0.020)
Diabetes (0,1)	0.070** (0.022)	0.071*** (0.022)	-0.087*** (0.025)	-0.069** (0.025)	0.011 (0.021)	0.007 (0.021)
Cancer (0,1)	0.047 (0.024)	0.046 (0.024)	-0.259*** (0.029)	-0.273*** (0.029)	-0.059* (0.024)	-0.055* (0.024)
IL-6	0.184*** (0.011)	0.184*** (0.011)	-0.034** (0.013)	-0.028* (0.013)	0.006 (0.011)	0.004 (0.011)
TNFR1	0.366*** (0.012)	0.366*** (0.012)	-0.061*** (0.015)	-0.062*** (0.014)	-0.064*** (0.012)	-0.064*** (0.012)
CMV Ab		-0.005 (0.009)		-0.130*** (0.010)		0.033*** (0.008)

* p < 0.05; ** p < 0.01; *** p < 0.001

Table S6. Mediation results for age, disease (HCVD = hypertension or CVD), cytokines, and naïve T cells in the US HRS (n = 8,261)

Mediation path	Prop. mediated	95% CI	p
Age→TNFR1→IL-10	100	100 – 100	<0.001
HCVD→IL-6→IL-10	47.9	24.5 – 100	0.014
HCVD→TNFR1→IL-10	74.2	52.5 – 100	<0.001
Diabetes→IL-6→IL-10	20.8	11.0 – 40.0	<0.001
Diabetes→TNFR1→IL-10	56.5	43.9 – 76.7	<0.001
Cancer→IL-6→IL-10	13.2	0.0 – 62.6	0.130
Cancer→TNFR1→IL-10	43.2	22.5 – 97.5	<0.001
Age→TNFR1→CD4T	10.1	5.7 – 15.0	<0.001
HCVD→IL-6→CD4T	12.1	0.0 – 100	0.180
HCVD→TNFR1→CD4T	30.7	0.0 – 100	0.084
Diabetes→IL-6→CD4T	3.6	0.7 – 10.0	0.014
Diabetes→TNFR1→CD4T	14.9	6.7 – 32.0	<0.001
Cancer→TNFR1→CD4T	2.3	1.0 – 4.0	<0.001
Age→TNFR1→CD8T	5.0	3.2 – 7.0	<0.001
Cancer→TNFR1→CD8T	9.6	3.7 – 31.0	0.002

Table S7. Regression models predicting IL-10 and GDF15 in the UKB. Cytokines were standardized, with resulting regression coefficients reflecting changes in standard deviations (n = 40,638). Estimates are reported as β (SE).

	IL-10	IL-10	GDF15	GDF15
Age (years)	0.002^{**} (0.001)	-0.004^{***} (0.001)	0.050^{***} (0.0005)	0.038^{***} (0.0004)
Male (0,1)	-0.025^{**} (0.009)	-0.017^{*} (0.009)	0.160^{***} (0.007)	0.125^{***} (0.006)
Hypertension or CVD (0,1)	0.032^{**} (0.010)	-0.026^{**} (0.010)	0.228^{***} (0.008)	0.109^{***} (0.007)
Diabetes (0,1)	0.134^{***} (0.021)	0.059^{**} (0.021)	1.050^{***} (0.017)	0.888^{***} (0.015)
Cancer (0,1)	0.063^{***} (0.018)	0.046^{**} (0.017)	0.078^{***} (0.014)	0.036^{**} (0.012)
IL-6		0.047^{***} (0.005)		0.104^{***} (0.004)
TNFR1		0.042^{***} (0.006)		0.309^{***} (0.004)
IL-1β		-0.006 (0.005)		-0.015^{***} (0.003)
TNF-α		0.237^{***} (0.006)		0.081^{***} (0.004)

Table S8. Logistic regression models predicting odds of hospitalization or death from COVID-19 in subsample without inflammatory diseases/conditions (n = 154 out of n = 17,665).

	Odds ratios with 95% confidence intervals		
Age (years)	1.06*** (1.03, 1.08)	1.04*** (1.02, 1.06)	1.03* (1.00, 1.05)
Male (0,1)	1.84*** (1.25, 2.44)	1.76*** (1.18, 2.33)	1.64** (1.10, 2.18)
IL-6		1.14 (0.94, 1.35)	1.06 (0.86, 1.26)
TNFR1		1.45*** (1.15, 1.76)	1.24 (0.96, 1.52)
IL-1β		0.91 (0.75, 1.07)	0.92 (0.76, 1.09)
TNF-α		1.10 (0.87, 1.34)	0.99 (0.77, 1.21)
IL-10			1.27** (1.07, 1.48)
GDF15			1.53*** (1.19, 1.88)

Listed diseases or conditions include hypertension or CVD, diabetes, cancer, COPD, asthma, or allergy. Cytokines were standardized, with resulting regression coefficients reflecting changes in standard deviations.

* $p < 0.05$; ** $p < 0.01$; *** $p < 0.001$

Table S9. Mediation results for age, diseases (HCVD = hypertension or CVD) and cytokines (n = 40,638)

Mediation path	Proportion mediated	95% CI	p
Age→IL-6→GDF15	2.2	1.8 – 2.5	<0.001
Age→TNFR1→GDF15	11.0	10.3 – 11.8	<0.001
Age→TNF-α→GDF15	1.2	1.0 – 1.5	<0.001
HCVD→IL-6→GDF15	13.6	11.5 – 16.0	<0.001
HCVD→TNFR1→GDF15	27.9	24.1 – 32.1	<0.001
HCVD→TNF-α→GDF15	1.9	0.7 – 3.2	0.002
Diabetes→IL-6→IL-10	8.1	4.2 – 20.9	<0.001
Diabetes→TNFR1→IL-10	14.7	7.9 – 36.5	<0.001
Diabetes→TNF-α→IL-10	13.1	1.7 – 35.0	0.034
Diabetes→IL-6→GDF15	1.3	0.9 – 1.7	<0.001
Diabetes→TNFR1→GDF15	8.0	7.0 – 9.2	<0.001
Cancer→IL-6→IL-10	4.1	1.0 – 15.1	0.012
Cancer→TNFR1→IL-10	5.6	2.5 – 16.1	0.006
Cancer→IL-6→GDF15	10.5	2.4 – 26.3	0.014
Cancer→TNFR1→GDF15	36.1	21.3 – 63.0	<0.001

Table S10. Mediation results for age, diseases (HCVD = hypertension or CVD), cytokines, and COVID-19 hospitalizations or deaths in the UKB (n = 40,638 with n = 552 hospitalized or died)

Mediation path	Proportion mediated	95% CI	p
Age→IL-6→COVID	6.8	3.6 – 13.0	<0.001
Age→TNFR1→COVID	16.2	10.1 – 27.7	<0.001
Age→GDF15→COVID	38.2	21.3 – 77.8	<0.001
HCVD→IL-6→COVID	12.0	6.0 – 33.8	<0.001
HCVD→TNFR1→COVID	16.4	8.6 – 37.9	<0.001
HCVD→GDF15→COVID	12.4	5.5 – 36.1	0.008
Diabetes→IL-6→COVID	8.3	2.7 – 53.0	0.026
Diabetes→TNFR1→COVID	26.7	13.3 – 1.00	0.014
Diabetes→GDF15→COVID	81.6	26.2 – 1.00	0.046
TNFR1→IL-10→COVID	2.2	0.5 – 5.9	0.008
TNFR1→GDF15→COVID	28.0	15.3 – 48.5	<0.001
IL-6→IL-10→COVID	2.9	0.6 – 7.3	0.008
IL-6→GDF15→COVID	14.9	7.5 – 28.1	<0.001

Table S11. Mediation results for age, cytokines, and COVID-19 hospitalizations or deaths in the UKB subsample without listed diseases (n = 17,665 total with n = 154 hospitalized or died)

Mediation path	Proportion mediated	95% CI	p
Age→TNFR1→COVID	12.1	4.3 – 26.2	<0.001
Age→GDF15→COVID	37.6	12.6 – 97.2	0.002
TNFR1→IL-10→COVID	5.0	0.9 – 36.0	0.036
TNFR1→GDF15→COVID	32.7	12.6 – 99.2	0.002
